# *De novo* mosaic and partial monosomy of chromosome 21 in a case with superior vena cava duplication

**DOI:** 10.1186/s13039-020-00513-2

**Published:** 2020-09-12

**Authors:** Abul Kalam Azad, Lindsay Yanakakis, Samantha Issleb, Jessica Turina, Kelli Drabik, Christina Bonner, Eve Simi, Andrew Wagner, Morry Fiddler, Rizwan Naeem

**Affiliations:** 1 Department of Pathology/Molecular Pathology, Montefiore Medical Center, Albert Einstein College of Medicine, 1635 Poplar St., Bronx, NY 10461 USA; 2 Insight Medical Genetics, Chicago, IL USA; 3 Chicago Women’s Health Group, Chicago, IL USA

**Keywords:** Chromosome 21, Partial monosomy, Mosaicism, Superior vena cava duplication

## Abstract

**Background:**

Full or partial monosomy of chromosome (chr) 21 is a very rare abnormal cytogenetic finding. It is characterized by variable sizes and deletion breakpoints on the long arm (q) of chr 21 that lead to a broad spectrum of phenotypes that include an increased risk of birth defects, developmental delay and intellectual deficit.

**Case presentation:**

We report a 37-year-old G1P0 woman initially screened by non-invasive prenatal testing with no positive findings that was followed by an 18-week anatomy scan with a fetal finding of duplication of the superior vena cava (SVC). The medical and family history was otherwise uneventful. After appropriate genetic counseling, amniocentesis was performed to evaluate suspected chromosomal anomalies.

**Conclusions:**

Interphase fluorescent in situ hybridization revealed loss of one chr 21 signal that was further delineated by chromosomal microarray analysis on uncultured amniocytes as a terminal 10 Mb deletion on chr 21q. Karyotype and microarrays on cultured amniocytes showed two cell lines for a mosaic 21q terminal deletion and monosomy 21. The combined molecular cytogenetics results reported following the ISCN 2016 guideline as mos 46,XX,del(21)(q22)dn[20]/45,XX,-21dn[10].nuc ish(D21S342/D21S341/D21S259x1)[100].arr[GRCh37] 21q11.2q22.12(15412676_36272993)x1~2,21q22.12q22.3(36431283_47612400)x1. Parental chromosomal analysis revealed normal karyotypes. Thus, this was a *de novo* mosaic full and partial monosomy of chr 21 in a case with SVC duplication. Despite the association of congenital heart disease with monsomy 21 we could not find any published literature or online databases for this cytogenetic abnormality. The patient terminated the pregnancy following the abnormal molecular cytogenetic results due to the possible challenges the baby would face if carried to term.

## Background

Monosomy 21 is a very rare cytogenetic anomaly caused by loss of chromosome (chr) 21 or deletion of variable regions of the long arm (q) of chr 21. To date only a handful of cases have been described the anomaly/condition with different terminology such as 21q deletion syndrome, 21q- syndrome or partial 21q monosomy. The severity of the phenotype depends on the location and size of the deleted region [1]. In general, the condition leads to an increased risk of birth defects, developmental delay and intellectual deficit. Proximal and distal deletions lead to milder phenotypes [2]. Conversely, deletions involving band 21q22 have a more severe effect on the phenotype. A complete monosomy 21 is not compatible with a live birth [1].

Non-invasive prenatal testing (NIPT) has been widely used to detect common fetal chromosome aneuploidies, such as trisomy 21, 18, and 13 [3]. Among them trisomy 21 is mostly compatible with life and causes Down syndrome. However, full or partial monosomy 21 is much rarer and not typically detected by NIPT screens. Only a few patients with such a finding have been reported [4–7]. The regions of the partial monosomy with associated phenotypes are variable and based on the deleted segments or deletion breakpoints [8].

## Clinical history

A 37-year-old G1P0 woman was referred to Insight Medical Genetics (Chicago, IL) for genetic counselling following an abnormal anatomic ultrasonography (USG) result at 18-weeks of gestation. The USG showed superior vena cava (SVC) duplication. NIPT (Harmony, Roche Diagnostics, IN) results were negative for trisomy 21, 18, and 13 with a reported fetal fraction of cell-free DNA of 5.6%.

The patient and her non-related partner were of European ancestry. There was no history of known genetic conditions or congenital anomalies nor a history of multiple miscarriages, familial intellectual disabilities, or other relevant medical conditions. Additionally, there was no known exposure to teratogens including alcohol or drugs during her pregnancy.

The patient was counseled by a genetic counselor regarding a possible association between the USG findings and chromosomal abnormalities including aneuploidy, microdeletions and microduplications. Prenatal diagnosis of chromosome abnormalities was offered, and the patient elected to have an amniocentesis. Interphase fluorescent in situ hybridization (FISH) (Cytocell Ltd., NY), karyotyping and comparative genomic hybridization (CGH) + single nucleotide polymorphism (SNP) microarray analysis of the amniocytes from the amniotic fluid was performed (SurePrint G3 Human CGH Microarray 4x180K, Agilent Technologies Inc., CA).

## Results

**Fig. 1:**
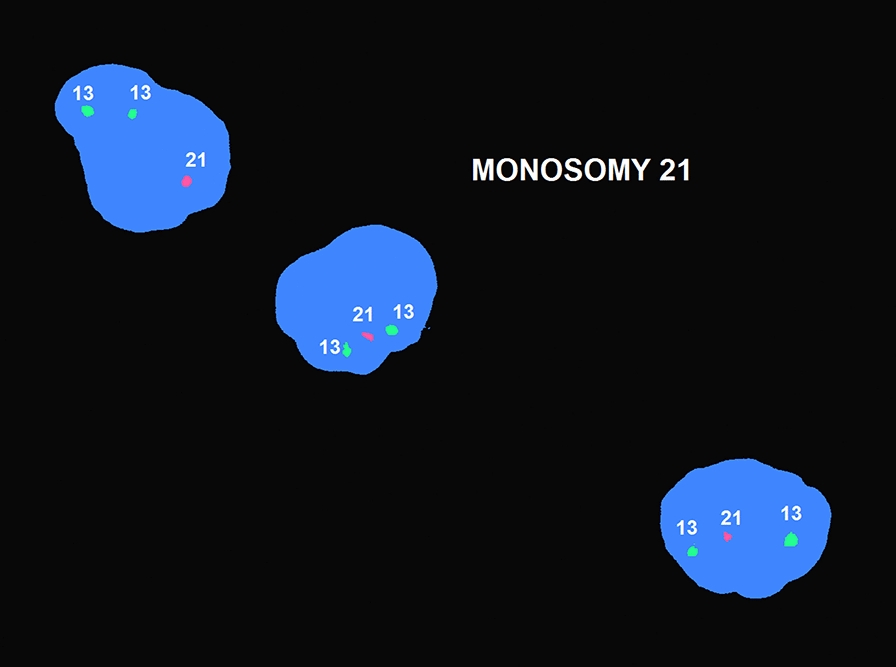
Interphase FISH analysis using LSI 13 DNA probes (RB1: 13q14) labeled with SpectrumGreen and LSI 21 SpectrumOrange probe (21q22.13-q22.2) revealed deletion of chr 21 in uncultured amniocytes

Interphase or nuclear in situ hybridization (nuc ish) on uncultured amniocytes revealed nuc ish(D21S342/D21S341/D21S259x1)[100]. Hybridization with a DNA probe localized to chr 21q22.13-q22.2 produced one signal (Fig. 1). The absence of one signal for chr 21 may indicate either a complete loss of chr 21 (monosomy 21), or a deletion/unbalanced translocation involving chr 21q22.13-q22.2.

**Fig. 2:**
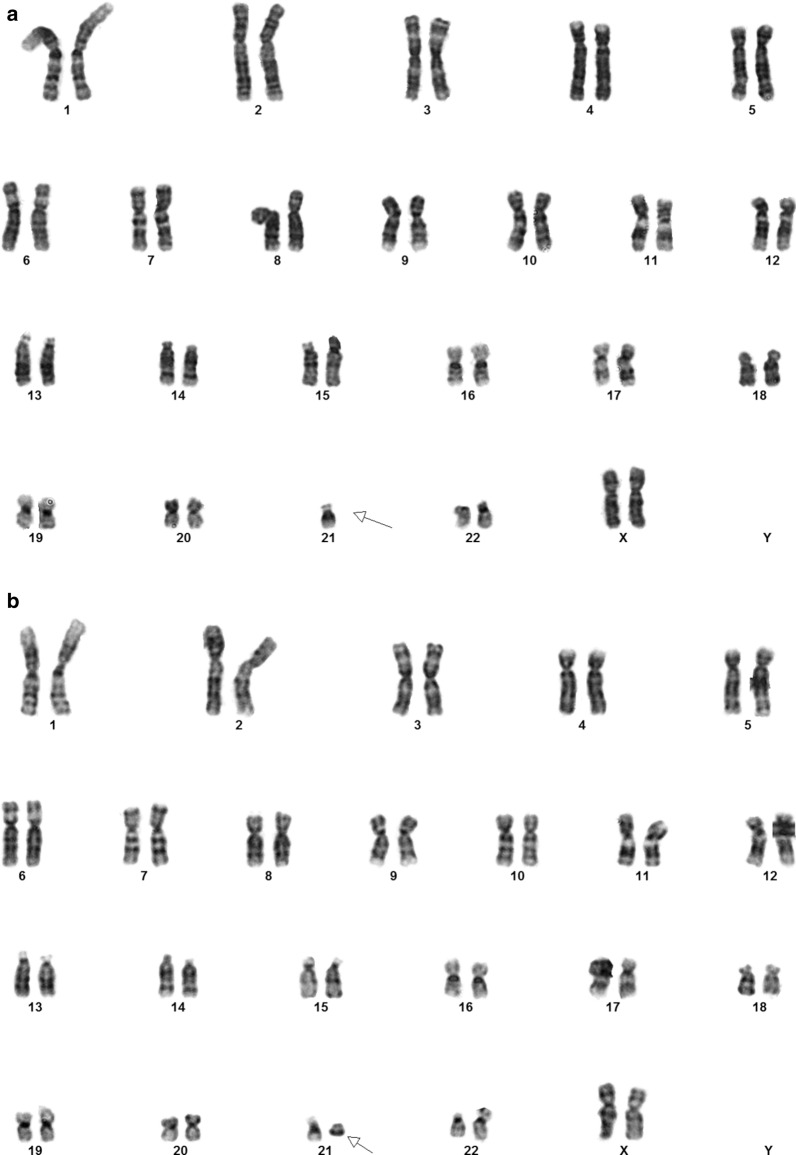
G-banded karyotype of the cultured amniocytes showed full and partial deletion of chr 21 in two different metaphases

Chromosome analysis on cultured amniocytes showed the presence of two abnormal cell lines: mos 46,XX,del(21)(q22)dn[20]/45,XX,-21dn[10]; see karyotype (Fig. 2). Of the total 30 cells examined from four independent cultures, 20 cells had 46 chromosomes with a loss of the distal part of the long (q) arm of chr 21 at band q22 and 10 cells showed one copy of chr 21 (monosomy 21). Parental chromosome analyses showed normal karyotypes. The parents did not have a rearrangement involving 21q22 and thus the deletion in this prenatal specimen was apparently *de novo* (dn) in origin. The two abnormal cell lines were observed in all four independent cultures and fit the definition of true mosaicism.

**Fig. 3:**
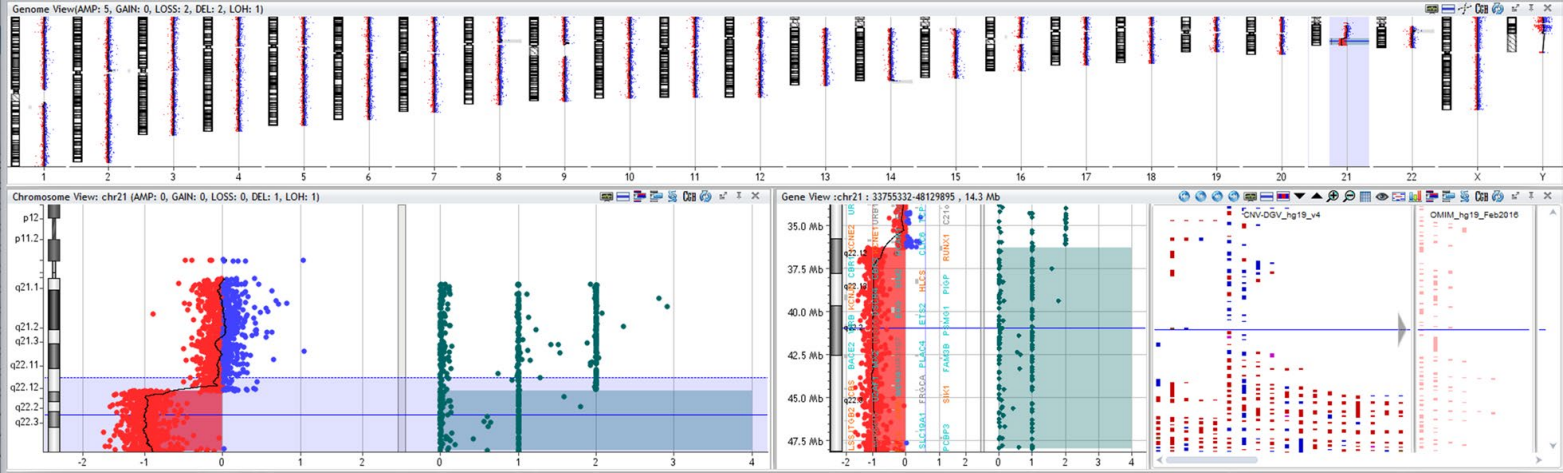
Chromosomal SNP microarray using DNA from uncultured amniocytes showing a terminal deletion of chr 21q22.12q22.3

CGH + SNP microarray analysis on uncultured amniocytes from amniotic fluid showed a pathogenic partial terminal deletion of 10 Mb on the long arm (q) of chr 21 (Fig. 3). This result confirmed the interphase FISH findings of non-mosaic loss of 21q22 with no evidence of complete loss of chr 21. The identified deletion is described as arr[GRCh37] 21q22.12q22.3(36285036_48090317)x1. Current evidence obtained from reputable databases and peer-reviewed literature indicated that this deletion is causative of partial chr 21q monosomy.

**Fig. 4:**
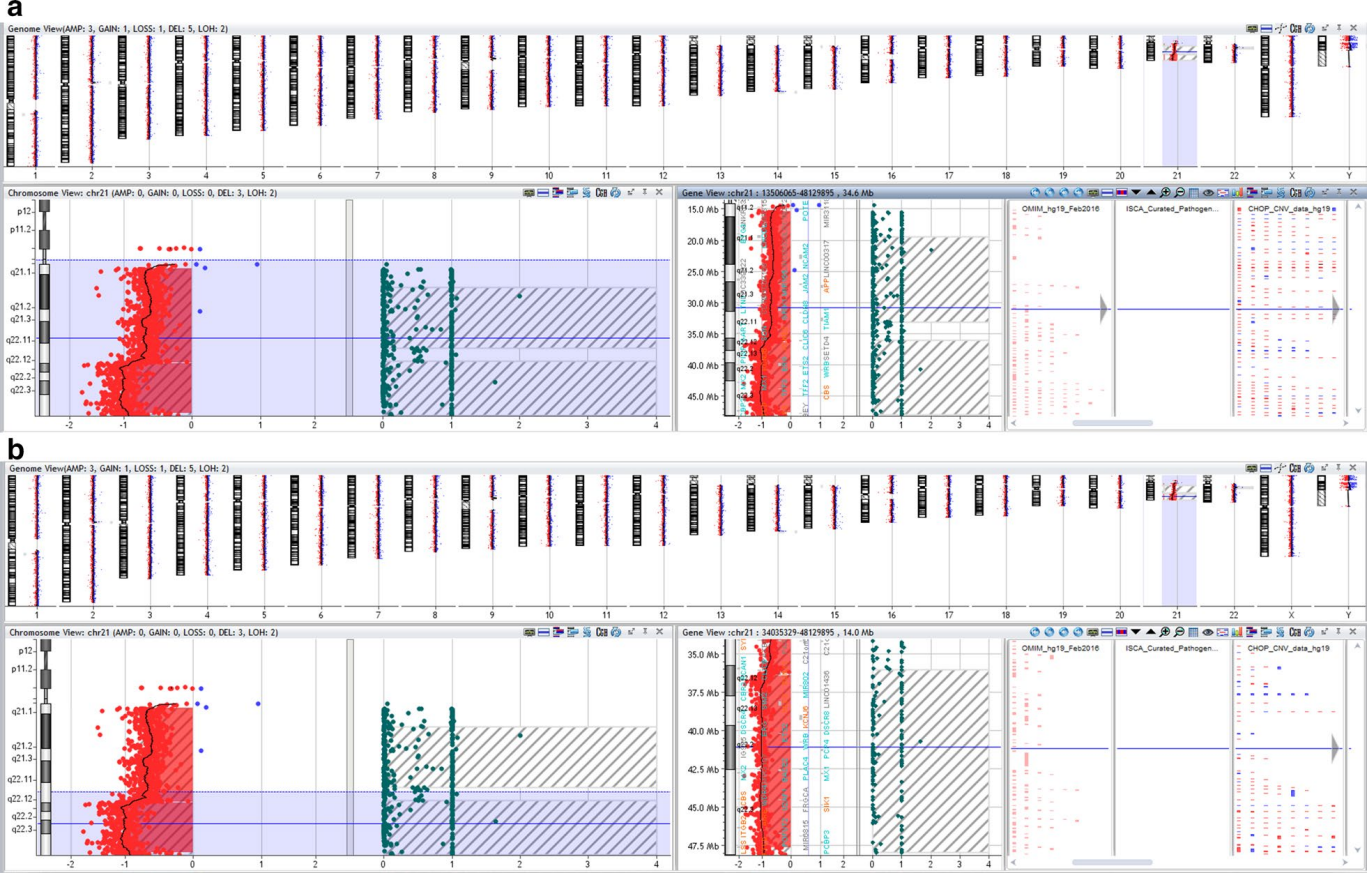
Chromosomal SNP microarray using DNA from cultured amniocytes showing the full and terminal deletion of chr 21q22.12q22.3. The B-allele frequency (BAF) representing the mosaic nature of this finding

Follow-up CGH + SNP microarray analysis on cultured amniocytes showed mosaicism for monosomy 21 and the partial chr 21q deletion (Fig. 4). The ISCN of the microarray is arr[GRCh37] 21q11.2q22.12(15412676_36272993)x1 ~ 2,21q22.12q22.3(36431283_47612400)x1. This result confirmed the karyotype finding of the cultured amniocytes.

## Discussion and conclusions

In this case report the fetus was first screened with NIPT which returned no indication of aneuploidy for the target chromosomes of that analysis. Ultrasonography at 18-weeks of gestation revealed a duplication of SVC. Following a subsequent amniocentesis, the fetus was determined to have mosaicism for both partial and full chr 21 deletion. The parents’ karyotypes were normal, suggesting this chromosomal 21 anomaly was *de novo* and the mosaic pattern was apparently of post-zygotic origin. Although the mother was 37 years old and thus considered to be of advanced maternal age, increased maternal age is not in itself an effective screen for aneuploidy [9]. In contrast, structural chromosomal abnormalities, including microdeletions and microduplications, do not increase in frequency with maternal age [10]. During genetic counseling the parents were informed about the recurrent risk of future pregnancies along with laboratory test artifacts and nondisjunction risk. The patient terminated the pregnancy following the abnormal molecular and cytogenetic results due to the possible challenges the baby would face if carried to full term or live birth.

Full monosomy of chr 21 is rare and most often is lethal in intra-uterine life. However, partial monosomy 21 is even rarer and only a few patients have been reported in the literature [11, 12]. Correlations between clinical phenotype and genotype were hard to determine due to the variability of reported deletion breakpoints [13]. Neonates with partial 21q deletions display multiple dysmorphic features at birth such as cardiac, pulmonary, renal, skeletal and genitourinary abnormalities. These infants often fail to thrive and if they did survive, they displayed intellectual disabilities, congenital malformations of the heart and several other physical disabilities and physiological disorders.

**Fig. 5:**
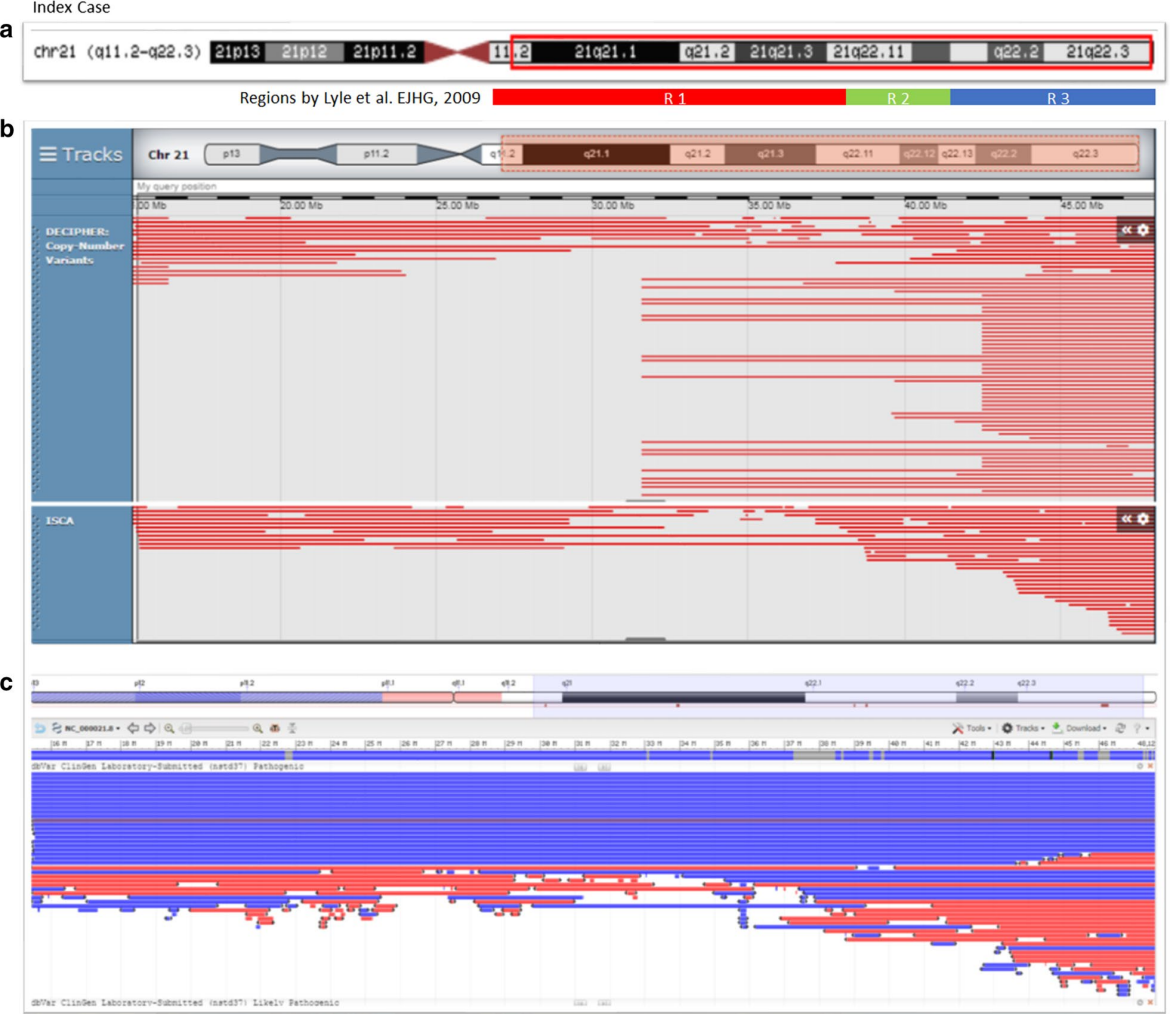
Bioinformatics analysis. **a** Index case and the deleted region at position: chr 21:15412676-48090317. **b** DECIPHER CNVs filter as follows: Loss only—pathogenic, Likely pathogenic, all sizes, squish; ISCA filter: Loss only—pathogenic, squish. **c** NCBI dbVar showing both gain and loss of pathogenic CNVs (Note: likely pathogenic is the same as pathogenic CNVs—see variant summary counts for nstd37 in dbVar)

Previous studies have suggested *KCNE1*, *RCAN1*, *CLC6*, *RUNX1* and *DYRK1A* as candidate genes for congenital cardiac anomalies residing on chr 21 [5, 6, 8]. It is highly anticipated that the loss of these genes on chr 21 contributed to the fetal cardiac phenotype of SVC duplication. Lyle et al. suggested three regions of chr 21q based on the genotype–phenotype correlations. These regions (Fig. 5) outlined as Region 1: ~31.2 Mb, most severe phenotype; Region 2: ~31.2–36 Mb, severe phenotype; and Region 3: 36–37.5 Mb, milder phenotype, respectively [12, 14, 15]. The terminal deletion on chr 21q was comparable in size (10 Mb) to the one we have identified in our index case. The cardiac defects described for monosomy chr 21 include pulmonary stenosis, patent ductus arteriosus and septal defects [1, 16]. Recently a case was reported of interrupted inferior vena cava with azygous continuation [17].

Bioinformatics analysis of this region through online databases were also performed. DECIPHER, ISCA and NCBI-dbVar databases were used to identify the loss of copy number variants and its phenotype [18]. There were only a few pathogenic or likely pathogenic variants reported for the chromosomal region we have identified through CGH + SNP microarray analysis (Fig. 5). Most of the patients reported have the terminal deletion of chr 21q. We reviewed only those cases that substantially overlapped with our microarray findings i.e. 21q11.2q22.12 and 21q22.12q22.3.

To delineate the specific phenotype of SVC duplication relating to chr 21q11.2q22.12 and chr 21q22.12q22.3 deletions we also searched NCBI-PubMed database. There were only 8 patients described in the literature with the condition of “congenital heart defect” with variable 21q breakpoints [5, 6]. However, we could not identify any mosaic patients that resemble the genotype–phenotype findings we investigated here. The present case report is the first to describe superior vena cava duplication as a constituent in the spectrum of cardiac anomalies found in monosomy chr 21. Thus, this clinical feature with the cytogenetics results add to the body of knowledge of understanding the molecular pathogenies of monosomy 21/partial 21q monosomy/21q deletion syndrome.

The clinical use of NIPT to screen high-risk patients for the most common fetal chromosomal aneuploidies has become increasingly common. The American College of Medical Genetics and Genomics (ACMG) and the American College of Obstetricians and Gynecologists (ACOG) recommended NIPT as a screening test for common trisomies (i.e. 21, 18, and 13) and, if requested, sex chromosomal aneuploidies as well [3, 19]. NIPT screening test does not eliminate the possibility of other anomalies of the tested chromosomes like mosaicism, deletions or duplications [20]. Therefore, USG at an appropriate gestational age should be performed and when fetal anomalies are detected, invasive diagnostic testing with chorionic villus sampling or amniocentesis, depending on gestational age, are recommended to detect those chromosomal abnormalities [3].

Despite negative NIPT results, the abnormal anatomy scan of the fetus in this report at 18-weeks of pregnancy prompted us to conduct further invasive diagnostic tests. The invasive diagnostic tests we performed were much more informative than the fetal cell-free DNA NIPT test. Results of interphase FISH and SNP + aCGH from uncultured amniocytes were similar; subsequent karyotyping and SNP + aCGH of cultured amniocytes were concordant. Full monosomy 21 was also found to be in agreement in interphase FISH and chromosome analysis. Complete monosomy cell lines in the uncultured amniocytes might have exist in a very lower frequency in compare to the complete monosomy cell lines. However, in the culture conditions full monosomy confers an advantage and hence detected by microarray. Another possibility is that the complete monosomy cell lines are culture artifact; this could not be ruled out. These molecular and cytogenetic results reinforced that a single test does not always lead to a decisive diagnosis. Accordingly, analysis of uncultured cells from amniocentesis should be the preferred cytogenetic technique. During post-test follow-up, genetic counselors should make it clear that these cytogenetic testing artifacts could modify the recurrent risk of certain chromosomal abnormalities.

In conclusion, full and partial monosomy chr 21 is presumed to be lethal during the antenatal period. The fetus identified with this condition required a thorough investigation using a combination of conventional and molecular-cytogenetic techniques to exclude any ambiguity and determine the pathogenesis of the genomic imbalance.

## Data Availability

The results of the clinical tests are available upon request from Dr. Morry Fiddler, PhD on reasonable ground.

## Abbreviations

chr: Chromosome
dn: *De novo*
SVC: Superior vena cava
NIPT: Non-invasive prenatal testing
FISH: Fluorescent in situ hybridization
SNP: Single nucleotide polymorphism
aCGH: Array comparative genomic hybridization
CNV: Copy number variation
db: Database
